# Arthroscopic resection of localized tenosynovial giant cell tumor in the deep infrapatellar bursa: a case report

**DOI:** 10.3325/cmj.2023.64.135

**Published:** 2023-04

**Authors:** Damjan Dimnjaković, Ivan Bojanić

**Affiliations:** 1 Department of Orthopedic Surgery, University Hospital Center Zagreb, Zagreb, Croatia; 2 School of Medicine, University of Zagreb, Zagreb, Croatia

## Abstract

Tenosynovial giant cell tumor (TGCT) is a rare disease characterized by the proliferation of the synovial membrane of a joint, tendon sheath, or bursa. TGCTs in joints are subdivided into the diffuse or localized type. The localized TGCT most frequently affects the knee and may occur in any knee compartment. The most common localization is the Hoffa’s fat pad, followed by the suprapatellar pouch and the posterior capsule. Here, we describe a case of a histopathologically proven TGCT of the knee, found in an unusual localization in the deep infrapatellar bursa, which was diagnosed by magnetic resonance imaging. The tumor was entirely arthroscopically resected. The patient had no further complaints following the operation, and there was no recurrence at the 18-month follow-up. Even though TGCT of the knee is uncommon, it should not be overlooked by orthopedic and trauma surgeons, and excision should be regarded as a reliable treatment option. The form of surgical treatment, either open or arthroscopic, should be determined based on a combination of the surgeon's preference and the best approach to the anatomical location of the disease.

Tenosynovial giant cell tumor (TGCT) is a rare disease characterized by the proliferation of the synovial membrane of a joint, tendon sheath, or bursa (1-4). Within a joint, it is usually found either as the diffuse type, affecting the synovium of the whole joint, or the localized type, presenting as a limited solitary mass of either pedunculated or, less commonly, sessile nodular outgrowth of the synovial membrane (1-4).

It is typically found within a single joint, with the knee being the most common localization (1-4). Although the localized TGCT can occur in any compartment of the knee, the most common localization is the Hoffa’s fat pad (2) followed by the suprapatellar pouch and the posterior capsule. This report presents an unusual localization of the localized TGCT in the deep infrapatellar bursa, which was successfully treated with arthroscopic resection.

## Case report

**Figure 1:**
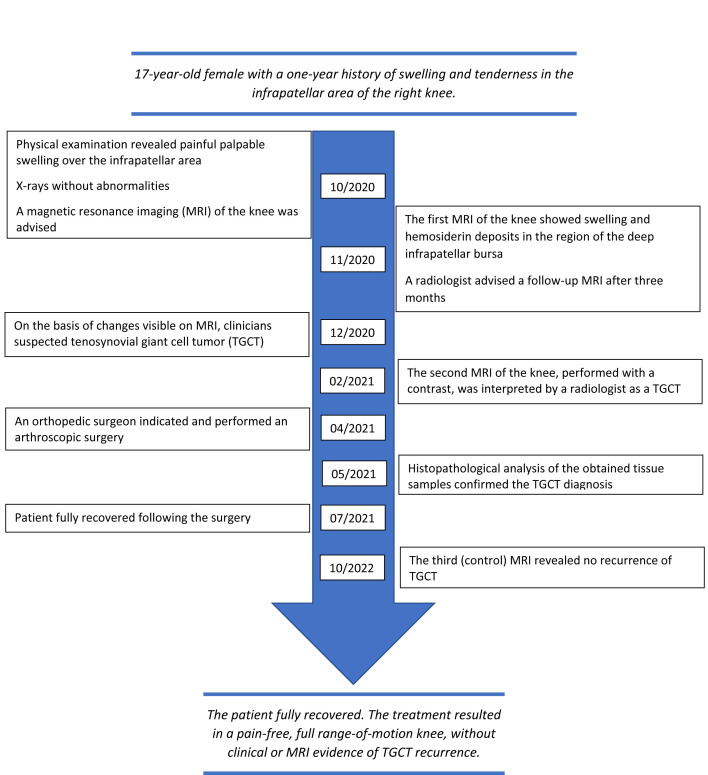
A timeline of relevant information from the patient’s history and this episode of care.

**Figure 2:**
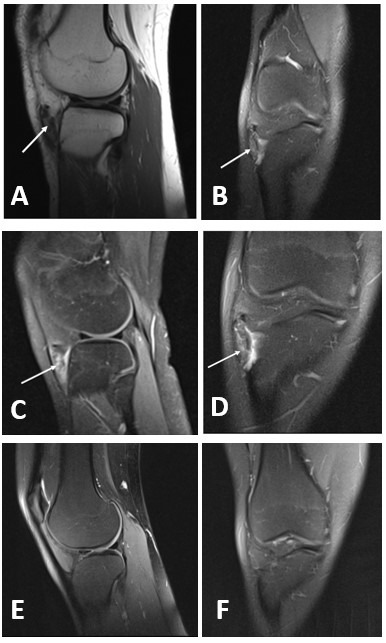
Preoperative and postoperative magnetic resonance images (MRI) of the right knee. (**A**) preoperative native MRI showing hemosiderin deposits in the area of the deep infrapatellar bursa (arrow) in a proton density and T2-weighted turbo spin-echo image in sagittal projection; (**B**) preoperative native MRI showing hemosiderin deposits in the area of the deep infrapatellar bursa (arrow) in a fat-suppressed proton-density-weighted turbo spin-echo image in coronal projection; (**C**) preoperative contrast-enhanced MRI showing proliferation of the synovium with signs of postcontrast imbibition, as well as hemosiderin deposits (arrow) in a T1-weighted spin-echo contrast-enhanced image in sagittal projection; (**D**) preoperative contrast-enhanced MRI showing proliferation of the synovium with signs of postcontrast imbibition, as well as hemosiderin deposits (arrow) in a fat-suppressed proton-density-weighted turbo spin-echo contrast-enhanced image in coronal projection; (**E**) postoperative MRI performed 18 months after surgery, showing no recurrence of the tumor in a proton-density-weighted turbo spin-echo image in sagittal projection; (**F**) postoperative MRI performed 18 months after surgery, showing no recurrence of the tumor in a proton-density-weighted blade fat-saturated image in coronal projection.

A 17-year-old female presented to our outpatient department with pain and swelling of the right knee (Figure 1). She mentioned having had a knee injury two years before, describing it as a direct trauma from falling on it. Physical examination revealed neither effusion within the knee joint nor restriction of knee motion. She had tenderness around the tibial tuberosity. Modest swelling was palpable over the infrapatellar area, especially on the lateral side. An x-ray showed no evidence of Osgood Schlatter's disease. At that time, the patient had already undergone an ultrasound examination, showing swelling in the area of the deep infrapatellar bursa. Magnetic resonance imaging (MRI) of the knee showed swelling and hemosiderin deposits in the region of the deep infrapatellar bursa (Figure 2A and 2B), but also suggested that the changes could be posttraumatic, and the radiologist recommended a follow-up MRI after three-months. After a thorough examination of the MRI images by the junior and senior author, the changes were considered highly suspicious of TGCT. The second MRI was conducted with both native and contrast-enhanced images (Figure 2C and 2D), now showing a proliferation of the synovium sized 3.5 × 0.7 × 1.7 cm with signs of postcontrast imbibition, as well as hemosiderin deposits. The radiologist then also considered the changes suggestive of TGCT, and operative excision was recommended. Due to the suitable size of the formation and the localization that enabled arthroscopic access, it was decided to perform the procedure arthroscopically.

**Figure 3:**
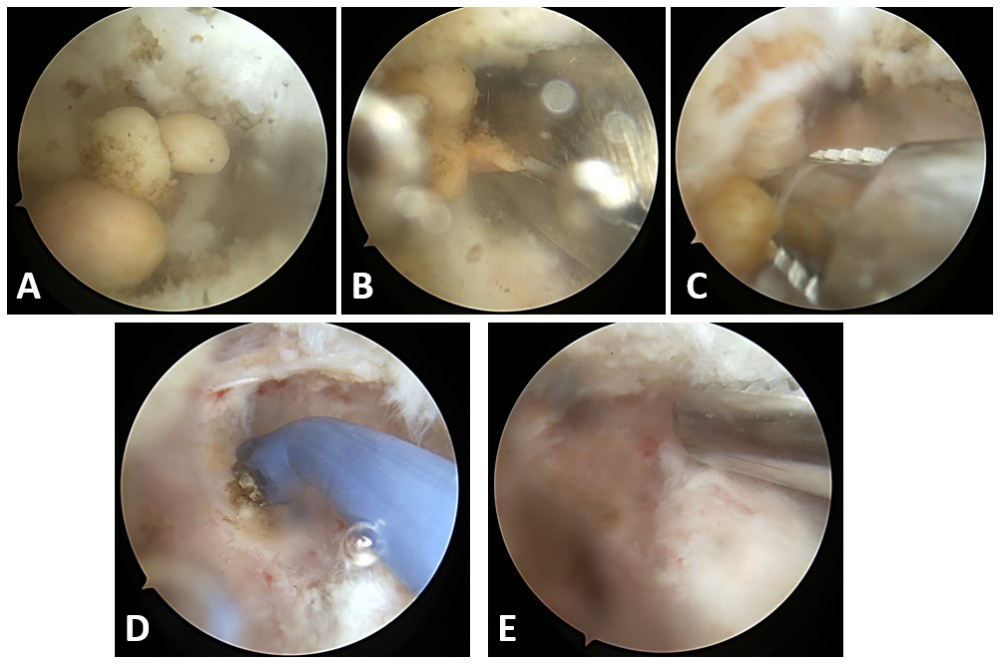
Arthroscopic images of removal of localized tenosynovial giant cell tumor in the deep infrapatellar bursa of the right knee. (**A**) multiple nodules of the pigmented, brownish synovia are found in the lateral part of the deep infrapatellar bursa; (**B**) arthroscopic grasper was used to take part of the tissue for histopathological analysis; (**C**) removal of the tumor with the arthroscopic shaver; (**D**) partial synovectomy was done after complete removal of the tumor with a radiofrequency ablation device; (**E**) arthroscopic image of the same area after complete removal and partial synovectomy.

The surgery was performed under spinal anesthesia with the patient in the supine position. A tourniquet was placed on the thigh but was not inflated during surgery. A high anterolateral portal and standard anteromedial were made. Diagnostic arthroscopy was performed in a systematic fashion, during which we did not identify any pathology. Then the meniscal anterior horns and intermeniscal ligament were visualized. By staying anterior to these structures, an anterior interval release was performed with the radiofrequency ablation device to approach the area between the patellar ligament and the anterior surface of the tibia. In addition, the knee was then extended to remove the tension from the patellar ligament, which facilitated the lowering of the arthroscope and the instrument into the area of the deep infrapatellar bursa. Multiple nodules of the pigmented, brownish synovia, typical of TGCT, were found in the lateral part of the deep infrapatellar bursa (Figure 3A). A part of the tissue was sent for histopathological analysis (Figure 3B), which later confirmed the diagnosis of TGCT. An arthroscopic shaver and a radiofrequency ablation device were used to completely excise the localized nodular mass (Figure 3C, D, and E). The perioperative period was uneventful. The patient fully recovered, with no pain or swelling at the final follow-up, 18 months after the surgery. The follow-up MRI revealed no recurrence of TGCT (Figure 2E and F).

## DISCUSSION

To our knowledge, this is the first report of arthroscopic resection of TGCT localized in the deep infrapatellar bursa and the second report of such localization (5). It also confirms the feasibility and success of arthroscopic resection of the localized TGCT in the knee (2,3). This localization makes the arthroscopic treatment technically demanding, similar to the arthroscopic resection of the residua of Osgood-Schlatter disease. In these cases, some authors even suggest an endoscopic approach to reach the interval posterior to the patellar ligament, while others suggest using x-ray during surgery (6-8). We managed to reach the interval and to perform total resection of the nodular mass by making a high anterolateral portal and by positioning the knee near full extension.

The ultimate goals of TGCT surgery are complete resection of the tumor, along with the achievement of clear margins (1-4). After Flandry et al (9) reported a successful arthroscopic removal of the localized TGCT from the knee in 1986, over time more and more patients have been operated on arthroscopically. A retrospective study conducted in 2017 on 100 patients with the localized TGCT in the knee reported no significant difference in recurrence between open and arthroscopic surgery (8.7% vs 9.1%) (2). Mastboom et al (3) reported a recurrence rate of 18% in patients in whom the localized TGCT was removed from the knee by an arthroscopic procedure (20 recurrences out of 114 procedures performed), compared with 9% after an open procedure (34 recurrences out of 400 procedures performed). There was a greater risk of recurrence when arthroscopic procedure was performed on a tumor greater than 5 cm in diameter (3). Based on these results, the authors (3) advised that arthroscopic resections be used for small/pedunculated and easily accessible lesions. When the lesion exceeds 5 cm in diameter, most arthroscopic resections are intralesional, potentially leaving residual disease in the joint, which can lead to recurrence (3). In both of these studies, a significantly higher number of complications was recorded after open surgery (88% vs 12%) (2,3).

Although this case report confirms the success of arthroscopic resection of the localized TGCT in the knee, we would still advise that the form of surgical treatment is determined based on the combination of the surgeon's preference and the best approach to the anatomical location of the disease.

## References

[1] Gouin F Noailles T Localized and diffuse forms of tenosynovial giant cell tumor (formerly giant cell tumor of the tendon sheath and pigmented villonodular synovitis). Orthop Traumatol Surg Res 2017 103 S91 7 10.1016/j.otsr.2016.11.002 28057477

[2] Patel KH Gikas PD Pollock RC Carrington RW Cannon SR Skinner JA Pigmented villonodular synovitis of the knee: A retrospective analysis of 214 cases at a UK tertiary referral centre. Knee 2017 24 808 15 10.1016/j.knee.2017.03.011 28442184

[3] Mastboom MJL Staals EL Verspoor FGM Rueten-Budde AJ Stacchiotti S Palmerini E Surgical treatment of localized-type tenosynovial giant cell tumors of large joints: a study based on a multicenter-pooled database of 31 international sarcoma centers. J Bone Joint Surg Am 2019 101 1309 18 10.2106/JBJS.18.01147 31318811

[4] Healey JH Bernthal NM van de Sande M Management of tenosynovial giant cell tumor: a neoplastic and inflammatory disease J Am Acad Orthop Surg Glob Res Rev 2020 4 e20.00028. 10.5435/JAAOSGlobal-D-20-00028 PMC7643913 33156160

[5] Kinsella SD Sennett BJ Carey JL Extra-articular pigmented villonodular synovitis of the deep infrapatellar bursa: a case report. JBJS Case Connect 2013 3 e133 10.2106/JBJS.CC.M.00165 29252289

[6] Klein W Endoscopy of the deep infrapatellar bursa. Arthroscopy 1996 12 127 31 10.1016/S0749-8063(96)90235-2 8838745

[7] Lui TH Endoscopic management of Osgood-Schlatter disease. Arthrosc Tech 2016 5 e121 5 10.1016/j.eats.2015.10.023 27073771 PMC4811213

[8] McDonough GR Rossi MJ Arthroscopic resection of symptomatic tibial tubercle ossicles for recalcitrant Osgood-Schlatter disease using a 2-portal technique. Arthrosc Tech 2022 11 e813 8 10.1016/j.eats.2021.12.041 35646564 PMC9134260

[9] Flandry FC Jacobson KE Andrews JR Localized pigmented villonodular synovitis of the knee mimicking meniscal injury. Arthroscopy 1986 2 217 21 10.1016/S0749-8063(86)80075-5 3801100

